# Nitric Oxide Enters Quorum Sensing via the H-NOX Signaling Pathway in *Vibrio parahaemolyticus*

**DOI:** 10.3389/fmicb.2019.02108

**Published:** 2019-09-18

**Authors:** Takahiro Ueno, Jonathan T. Fischer, Elizabeth M. Boon

**Affiliations:** ^1^Department of Chemistry, Stony Brook University, Stony Brook, NY, United States; ^2^Institute of Chemical Biology & Drug Discovery, Stony Brook University, Stony Brook, NY, United States

**Keywords:** nitric oxide, quorum sensing, H-NOX, histidine kinase, *Vibrio*

## Abstract

Nitric oxide (NO) plays a major role in the regulation of mammalian biological functions. In recent years, NO has also been implicated in bacterial life cycles, including in the regulation of biofilm formation, and the metabolism of the bacterial second messenger signaling molecule cyclic-di-GMP. In a previous study, we reported the discovery of an NO-responsive quorum sensing (QS) circuit in *Vibrio harveyi*. Here, we characterize the homologous QS pathway in *Vibrio parahaemolyticus*. Spectroscopic analysis shows *V. parahaemolyticus* H-NOX is an NO sensory protein that binds NO in 5/6-coordinated mixed manner. Further, we demonstrate that through ligation to H-NOX, NO inhibits the autophosphorylation activity of an H-NOX-associated histidine kinase (HqsK; H-NOX-associated quorum sensing kinase) that transfers phosphate to the Hpt (histidine-containing phosphotransfer protein) protein LuxU. Indeed, among the three Hpt proteins encoded by *V. parahaemolyticus*, HqsK transfers phosphate only to the QS-associated phosphotransfer protein LuxU. Finally, we show that NO promotes transcription of the master quorum sensing regulatory gene *opaR* at low cell density.

## Introduction

Quorum sensing (QS) is a cell-to-cell communication system utilized by bacteria to assess their population density and to coordinate population-wide changes in gene expression. Bacteria produce, secrete, and detect small signaling molecules called autoinducers (AI). Many types of autoinducers have been identified, some that are unique to a particular species, and some that are shared by multiple bacterial species (Eberhard et al., 1981; Engebrecht and Silverman, 1984; Henke and Bassler, 2004; Miller et al., 2004). Detection of AIs by a receptor protein in a QS pathway ultimately leads to changes in gene expression (Miller and Bassler, 2001). A classic example of a QS system is the well-studied LuxI-LuxR system found in several *Vibrio* species as well as other gram-negative bacteria (Miller and Bassler, 2001). In this system, LuxI synthesizes a homoserine lactone AI. This AI binds to the transcriptional regulator LuxR, which regulates the transcription of the *luxICDABE* operon. In addition to the LuxI-LuxR QS system, alternative QS circuits have also been characterized. Many QS systems in *Vibrio* species consist of an AI synthase, a corresponding AI-sensing histidine kinase (HK), and a cytoplasmic response regulator (RR). The QS-associated HKs in many bacteria are hybrid histidine kinases containing both kinase and receiver domains. These kinases typically have both kinase and phosphatase activities, allowing them to both phosphorylate and remove phosphate from the cognate response regulator or, as an intermediary, the histidine-containing phosphotransfer protein (Hpt) (Aiba et al., 1989; Makino et al., 1989; Tanaka et al., 1991). Frequently, the response regulators are transcription factors whose activity is modulated by phosphorylation (Hoch, 2000; Galperin, 2006; Gao et al., 2007).

Quorum sensing controls gene expression patterns as a function of population density. At low cell density, AI concentrations are also low and therefore the HKs are not complexed with the AI. Under these conditions, kinase activity predominates, and phosphate is transferred to the RR. The phosphorylated RR then facilitates changes in gene expression patterns, resulting in an adaptive cellular response. At high cell density, elevated AI concentrations drives HK binding to the AI, which switches the function of the kinase to act predominantly as a phosphatase. This ultimately leads to the removal of phosphate from the RR and causes corresponding changes in gene expression (Waters and Bassler, 2005; Bassler and Losick, 2006; Boyer and Wisniewski-Dyé, 2009).

*Vibrio parahaemolyticus* is a marine bacterium widely distributed in sea water around the world (McCarter, 1999; FAO/WHO, 2011), and is the leading cause of seafood-borne illnesses (Yeung and Boor, 2004; McLaughlin et al., 2005). *V. parahaemolyticus* produces a number of virulence factors, including the type III secretion system 1 (T3SS1), that are regulated by QS (Gode-Potratz and McCarter, 2011). *V. parahaemolyticus* shares a QS architecture with *Vibrio harveyi*, which is composed of three AIs (AI-2, HAI-1, and CAI-1), their synthases, and cognate membrane-bound sensory histidine kinases (LuxM/N, LuxS/PQ, and cqsA/cqsS, respectively) (Henke and Bassler, 2004; Ng and Bassler, 2009). All three AI-sensing kinases engage in phosphotransfer with LuxU, a histidine-containing phosphotransfer protein (Hpt). LuxU transfers phosphate to the response regulator LuxO, a transcription factor that regulates the transcription of quorum regulatory RNAs (*qrrs*) (Ng and Bassler, 2009). Along with RNA-binding protein Hfq, these *qrrs* regulate the translation of two master QS regulatory proteins, AphA, and OpaR. AphA and OpaR are both transcription factors that regulate the expression of various genes, including many involved in motility, surface sensing, biofilm formation, and virulence (Gode-Potratz and McCarter, 2011; Zhang et al., 2012; van Kessel et al., 2013; Kalburge et al., 2017).

A recent study conducted in our laboratory identified a fourth arm in the *V. harveyi* QS circuit. In this pathway, the gaseous signaling molecule nitric oxide (NO) is integrated into QS via ligation to the hemoprotein H-NOX and its partner, an H-NOX-associated hybrid HK called HqsK (H-NOX-associated quorum sensing kinase) (Henares et al., 2012). Membrane-permeable NO is detected by H-NOX in the cytoplasm. The ligation state of H-NOX regulates the activity of HqsK. When NO is not present, HqsK functions as a kinase, transferring phosphate to LuxO via LuxU. Upon NO binding to H-NOX, HqsK switches its activity from a kinase to a phosphatase, causing a reverse in phosphate flow and contributing to dephosphorylation of LuxO (Henares et al., 2012). In this work, we further characterize the NO-responsive QS circuit in the pathogenic marine bacterium *V. parahaemolyticus*.

## Materials and Methods

Unless otherwise noted, all the reagents were purchased in their highest available qualities and used as received.

### Bacterial Strains and Culture Method

*Escherichia coli* strain DH5α was used for cloning and *E. coli* BL21(DE3)pLysS was used for protein expression. DH5α was cultured in LB medium supplemented with 100 μg/mL ampicillin. BL21 (DE3) pLysS was cultured in 2XYT media (16 g/L Tryptone, 10 g/L yeast extract, and 5 g/L NaCl) supplemented with 100 μg/mL ampicillin and 34 μg/mL chloramphenicol. Both cultures were grown at 37°C with 250 rpm agitation. At A_600__nm_ between 0.6 and 0.9, protein expression was induced with 100 μM isopropyl-β-D-thiogalactopyranoside (IPTG) for 15 h at 16°C, then cells were harvested. *V. parahaemolyticus* strain EB101 (ATCC17802) was purchased from American Type Culture Collection and was grown by following the supplier’s culture method. In *V. parahaemolyticus* growth curve and qPCR experiment, an overnight culture of *V. parahaemolyticus* was diluted 1:500 into fresh media (BD234000 Nutrient broth with 3% NaCl), supplemented with 100 μg/mL ampicillin with various DETA NONO concentrations (Cayman Chemical). Cultures were grown at 37°C with agitation at 250 rpm. Bacterial growth was monitored by measuring A_600__nm_ and harvested at designated ODs.

### Molecular Cloning and Mutagenesis

The genome of *V. parahaemolyticus* strain EB101 has not been sequenced. Instead, we referred to the genome sequence of *V. parahaemolyticus* strain RIMD 2210633 for gene annotations and primer designing. *V. parahaemolyticus* genomic DNA was extracted using Zymo Research Quick-gDNA MiniPrep (D3006), by following the manufacturer’s instructions. Extracted *V. parahaemolyticus* gDNA was used as a template to amplify *Vp* H-NOX (VP1877, gene ID: 1189384), HK (VP1876 gene ID: 1189383), LuxU (VP2098, gene ID: 1189609), and Hpt proteins (VP1472, gene ID: 1188978 and VP2127, gene ID: 1189639) by PCR. Primers for VP1877, VP1876 kinase domain only, VP1876 internal kinase domain only and VP2098 contained *Nde*I and *Xho*I up-stream/down-stream restriction sites, respectively. VP1472 and VP2127 primers contained *Nde*I and *Not*I restriction sites, respectively. For all PCR reactions, Phusion High-Fidelity DNA Polymerase (New England Biolabs, M0530S) was used. Amplified products were double digested then ligated into pET-20b(+) vector (Novagen) or pET-23aHis-TEV and sequenced (Stony Brook DNA sequencing facility). Site-directed mutagenesis to generate VP1876 HK H214A and D499A was carried out following the QuikChange Site-directed Mutagenesis kit protocol (Stratagene). The primer sequence used for cloning and site-directed mutagenesis will be provided upon request.

### Protein Expression and Purification

All proteins contained a 6× His tag on either the N or C-terminus and were purified by immobilized metal ion affinity chromatography using Ni-NTA agarose. Protein concentrations were determined by Bradford assay with bovine serum albumin as a standard (Bradford, 1976).

### H-NOX Complex Preparation and Electronic Microscopy

In an anaerobic glove bag, purified *Vp* H-NOX protein was incubated with 10 mM potassium ferricyanide for 5 min to make Fe (III) H-NOX. Potassium ferricyanide was then removed using PD10 desalting column (GE Healthcare). Prepared *Vp* Fe (III) H-NOX was incubated with 20 mM sodium dithionite for 30 min then desalted to prepare Fe (II) H-NOX. Fe (II) H-NOX was further incubated with 3 mM DPTA NONOate (Cayman Chemicals) for 30 min then desalted to prepare Fe (II) NO⋅H-NOX. To make CO bound H-NOX, Fe (II) H-NOX was bubbled with CO for 10 min in a closed Reacti-Vial (Thermo Fisher Scientific). Electronic spectra of all samples were measured by a Cary 100 UV-Vis spectrophotometer (Agilent) equipped with Cary temperature controller set at 20°C. For temperature dependent 5, 6-coordinate NO⋅H-NOX distribution analysis, the sample’s temperature was varied from 4 to 40°C utilizing the temperature controller.

### NO Dissociation Rate Constant

This procedure has been described previously (Boon et al., 2006). Briefly, in an anaerobic glove bag, NO bound *Vp* Fe (II) H-NOX was diluted in 40 mM Tris-Cl, 150 mM KCl, 4 mM DTT, and 10% glycerol buffer at pH 8.0, then was rapidly mixed with an equal amount of the same buffer at 3, 30 or 300 mM of sodium dithionite saturated with CO. The absorption spectra were obtained by Cary 100 UV-Vis spectrophotometer (Agilent) equipped with a Cary temperature controller set at 20°C. NO dissociation was monitored by tracking increasing Fe (II) CO peak at 424 nm and decreasing Fe (II) NO peak at 399 nm. The resulting data was fitted to a two-phase exponential association equation, y = y^0^ + A_1_^∗^(1–e^–x/t1^) + A_2_^∗^(1–e^–x/t2^) to determine the NO dissociation rate constant. The experiments were repeated a minimum of three times for each sodium dithionite concentration. The resulting average *k*_off_ (NO) rate constants were reported with standard error of the mean (SEM). The dissociation rate constants were independent of sodium dithionite concentrations.

### HK Autophosphorylation Assay

[γ-^32^P]-ATP (6000 Ci/mmol, 10 mCi/mL) was purchased from PerkinElmer Health Sciences Incorporated. All reactions were performed at room temperature. Reaction mixtures contained final concentrations of 40 mM Tris-Cl, 150 mM KCl, 4 mM DTT, 10% glycerol, 4 mM MgCl_2_, 5 μM histidine kinase at pH 8.0. Histidine kinase in the reaction buffer was allowed to equilibrate at room temperature for a few minutes then reactions were initiated by the addition of ATP (2 mM) with trace amount of [γ-^32^P]-ATP (10 μCi). For SDS-PAGE analysis, reactions were stopped by adding 5× SDS loading dye (0.25% bromophenol blue, 0.5 M dithiothreitol, 50% Glycerol, 10% sodium dodecyl sulfate, 0.25 M, pH 6.8 Tris-Cl) at indicated time points. Quenched reactions were separated by SDS-PAGE. After gel drying, the sample radioactivity was detected by a Typhoon scanner (Typhoon 9400, Amersham Biosciences) then quantified with image processing software ImageJ. For the dot blot assay, reactions were quenched with 25 mM H_3_PO_4_, and 30 μL aliquots were pipetted onto a nitrocellulose membrane in a dot blot apparatus as described previously (Fischer et al., 2017). The membrane was washed with 25 mM H_3_PO_4_ and dried before exposure to a storage phosphor screen. Radioactivity in each spot was detected by a Typhoon scanner and quantified with ImageJ.

### HK Phosphatase Activity Assay

Purified *Vp* HK was mixed with the final concentration of 250 μM 3-O-methyl fluorescein phosphate (OMFP) in the reaction buffer (40 mM Tris–HCl, 150 mM KCl, and 10% glycerol at pH 8.0) at room temperature. HK’s phosphatase activity was quantified by measuring the production of OMFP hydrolysis product, O-methylfluorescein (OMF) at 450 nm. All data acquisition was done by VICTOR X5 Multilabel Plate Reader (PerkinElmer).

### HK/LuxU Phosphotransfer Assay

The components of the reaction mixture were the same as those used in the HK autophosphorylation assay. Reactions were performed at room temperature. The final concentration of 9.4 μM HK in the reaction buffer was incubated with ATP/[γ-^32^P]-ATP mix solution for 40 min to autophosphorylate HK. The final concentration of 40.7 μM LuxU was then added to the reaction mixture to initiate phosphotransfer. Reactions were quenched by the addition of 5× SDS loading dye at various time points followed by gel electrophoresis, gel drying and image scanning. Histidine kinase only with ATP mix solution, LuxU only with ATP mix solutions were run along with HK + LuxU as controls.

### HK Autophosphorylation Activity Inhibition by H-NOX

All reactions were carried out at room temperature. The components of the reaction mixture were the same as those used in the HK autophosphorylation assay. In an anaerobic glove bag, different oxidation/ligation states of H-NOX [final concentration 69.4 μM or varying (NO⋅H-NOX) for the titration] were incubated with *Vp* HK (D499A, final concentration 5.3 μM) for 30 min. Reactions were initiated by the addition of ATP/[γ-^32^P]-ATP mix solution. Thirty minutes after the initiation of the reaction, reactions were quenched by adding 5× SDS loading dye, followed by SDS-PAGE, gel drying and image scanning.

### Phosphotransfer Profiling

The procedure followed what’s been described by Laub (Laub et al., 2007). All reactions were performed at room temperature. The components of the reaction mixture were the same as those of the HK autophosphorylation assay. Final concentrations of 3.3 μM HK kinase domain and 3.3 μM internal kinase receiver (IKR) domain were incubated with ATP/[γ-^32^P]-ATP mix solution for 60 min. Final concentrations of 33 μM histidine containing phosphotransfer proteins (VP1472, VP2098, or VP2127) were added to the reaction mixtures to initiate phosphotransfer. At various time points, reactions were quenched by adding 5× SDS loading dye. Proteins were separated by gel electrophoresis followed by gel drying and image scanning.

### Phosphotransfer Specificity Test

All reactions were carried out at room temperature and the components of the reaction mixtures were the same as those used in phosphotransfer profiling. Reaction mixtures with various components, KD only, IKR only, VP1472 only, VP2098 (LuxU) only, VP2127 only, KD + IKR, KD + IKR + VP2098 (LuxU), KD + VP1472, KD + VP2098 (LuxU), and KD + VP2127, were incubated with ATP/[γ-^32^P]-ATP mix solution for 60 min. Reactions were quenched by the addition of 5× SDS loading dye, followed by gel electrophoresis, gel drying and image scanning.

### RNA Extraction, cDNA Synthesis, and qPCR

RNA was extracted from bacterial cultures using the PureLink RNA Mini Kit (Ambion). Extracted RNA was treated with dsDNase (Thermo Fisher Scientific). cDNA synthesis and qPCR were carried out using either DyNAmo SYBR Green 2-Step qRT-PCR Kit or Maxima First Strand cDNA Synthesis Kits for RT-qPCR and DyNAmo HS SYBR Green qPCR Kit (Thermo Fisher Scientific). qPCR was carried out using *secY* (VP0277) as a reference gene. Data was analyzed by ΔΔCT method (BIO-RAD). Sequences for primer sets used are as follows:

*aphA* forward: TCAGCGAAACTTATGGCTTG
*aphA* reverse: GTTGAAGGCGTTGCGTAGTA
*opaR* forward: GAAATTGCGCAAGTGTCTGT
*opaR* reverse: ACGGACAACATGGTTGAGAA
*secY* forward: CAGTGGTTTGGTCAGAATGG
*secY* reverse: GGGCTAAGAGCCAAAGACAC

## Results and Discussion

### *Vp* H-NOX Is an NO-Binding Protein

**FIGURE 1:**
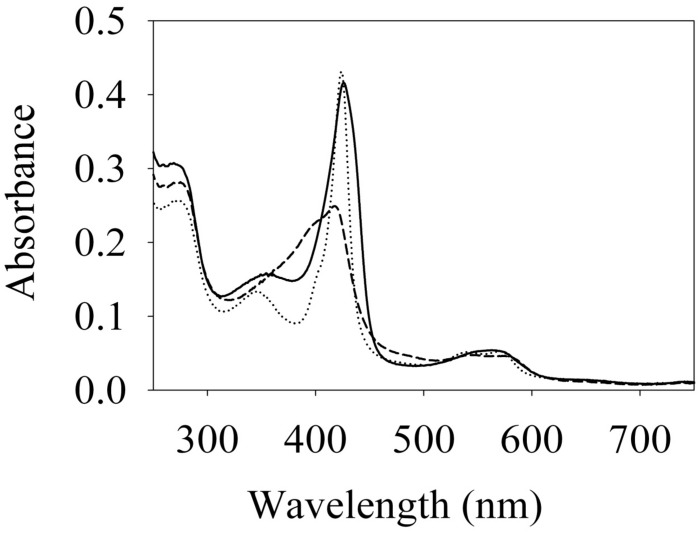
Absorption spectra of *Vibrio parahaemolyticus*. Fe (II) (–), Fe (II)⋅CO (⋅⋅⋅) and Fe (II)⋅NO (– – –) H-NOX at 20°C. Their Soret peaks are 426, 424, and 399/417 nm, respectively.

**FIGURE 2:**
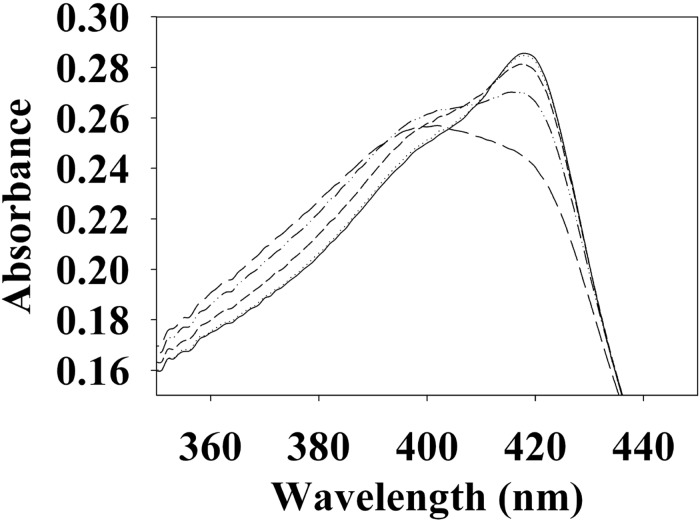
Temperature dependent absorption spectra of *V. parahaemolyticus* Fe^II^-NO H-NOX. The absorption spectrum shows the shift in the 5/6-coordinate Fe^II^-NO H-NOX ratio by temperature, lower temperatures favoring 6-coordinate (417 nm) and higher temperatures favoring 5-coordinate (399 nm). Absorption spectra were taken at 4°C (–), 10°C (⋅⋅⋅), 20°C (- - -), 30°C (–⋅⋅–), and 40°C (– – –).

**FIGURE 3:**
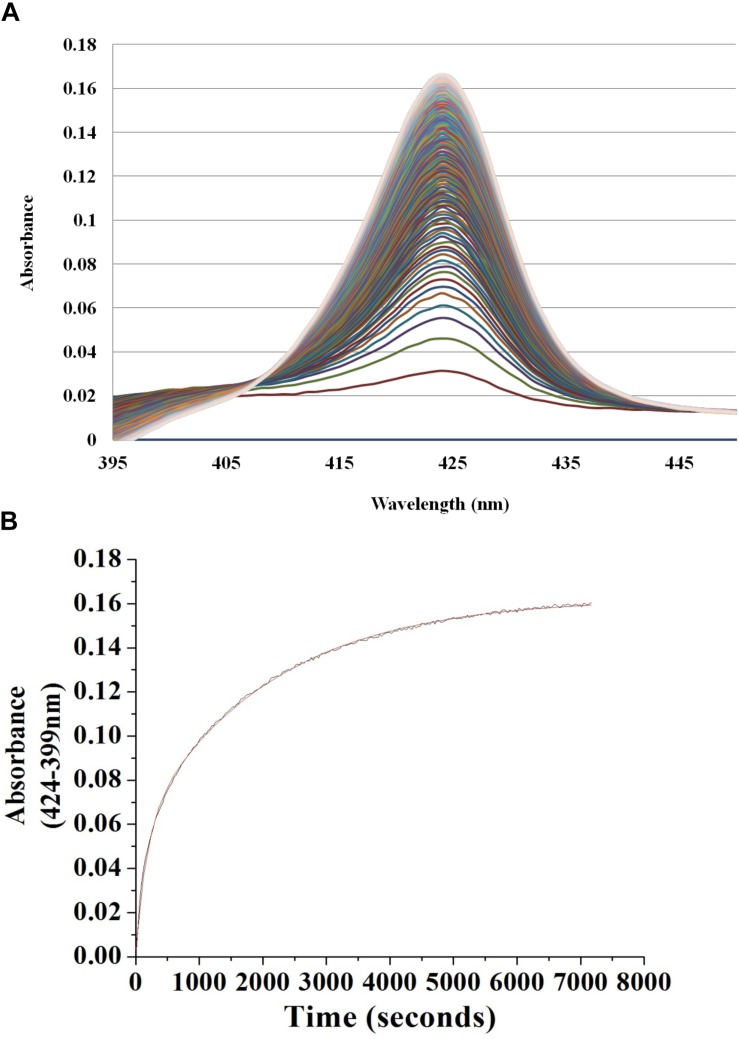
Continuous absorbance spectra during *Vp* H-NOX Fe^II^-NO dissociation rate constant determination. **(A)** Absorption spectra of *Vp* H-NOX Fe^II^-NO over time in the presence of saturating CO and Na_2_S_2_O_4_ as a released NO trap. **(B)** Absorbance difference spectrum shows the absorbance difference over time along with the exponential fit of the data.

To begin investigating the NO/H-NOX-mediated QS circuit in *V. parahaemolyticus*, we cloned, expressed, and purified *Vp* H-NOX (VP1877, gene ID: 1189384) and analyzed its spectroscopic and ligand-binding properties (Figure 1 and Table 1). The absorption peaks of purified H-NOX from *V. parahaemolyticus* in various oxidation and ligation states are similar to those of the eukaryotic H-NOX homolog sGC, as well as *V. harveyi*, and other previously characterized H-NOX proteins (Stone and Marletta, 1994; Karow et al., 2004; Boon et al., 2006; Henares et al., 2012). The reduced-unligated (Fe^II^-unligated) and CO-bound (Fe^II^-CO) *Vp* H-NOX complexes have Soret band maxima at 426 nm and 424 nm, respectively (Figure 1). Interestingly, NO-bound (Fe^II^-NO) *Vp* H-NOX has two Soret peaks, at 399 and 417 nm, which indicates the protein is a mixture of 5- and 6-coordinate heme at 20°C. In previous work on the H-NOX from *Nostoc punctiforme*, it was shown that the distribution of the Fe^II^-NO coordination state is temperature dependent, with lower temperatures favoring the 6-coordinate complex and higher temperature favoring the 5-coordinate state. We tested if this was also the case for *Vp* H-NOX by varying the temperature from 4 to 40°C. Indeed, we observed that the distribution of the 5- and 6-coordinate complexes was temperature dependent, being predominately 6-coordinate at 4°C with a shift toward 5-coordinate as the temperature was increased (Figure 2). This suggests that like *Np* H-NOX, a thermal equilibrium exists between 5- and 6- coordinate NO-bound *Vp* H-NOX, and cleavage of the Fe-His bond upon NO binding is temperature dependent. We also determined the NO dissociation rate constant for *Vp* H-NOX using a sodium dithionite/carbon monoxide trap (Figure 3; Kharitonov et al., 1997; Boon et al., 2005). We determined *k*_off_(NO) to be (4.3 ± 0.5) × 10^–4^ s^–1^ at 20°C, indicating a slow NO dissociation rate constant, similar to those of other previously characterized H-NOX proteins (Table 1). All of these results support that *Vp* H-NOX is an NO-binding protein.

**TABLE 1:** Electronic absorption spectra Soret peaks and NO dissociation rate constants of various H-NOX proteins.

**Protein**	**Soret peak (nm)**			**K_off_ (NO)**	**References**
	**Fe^II^-unligated**	**Fe^II^-CO**	**Fe^II^-NO**	**× 10^–4^ s^–1^**	
sGC^a^	431	423	398	3.6 ± 0.8	Henares et al., 2012
*Tt* H-NOX^b^	431	424	420	5.6 ± 0.5	Bradford, 1976
*Vp* H-NOX	426	424	399/417	4.3 ± 0.5	This work
*Vh* H-NOX^c^	429	423	399	4.6 ± 0.9	Kalburge et al., 2017
*Np* H-NOX^d^	430	423	400/416	–	Boon et al., 2006

*^*a*^H-NOX domain from soluble guanylate cyclase from bovine lung. ^*b*^H-NOX from *Thermoanaerobacter tengcongensis*. ^*c*^H-NOX from *Vibrio harveyi*. ^*d*^H-NOX from *Nostoc punctiforme*.*

### *Vp* HqsK Is an Active Hybrid Kinase With Kinase and Phosphatase Activities

**FIGURE 4:**
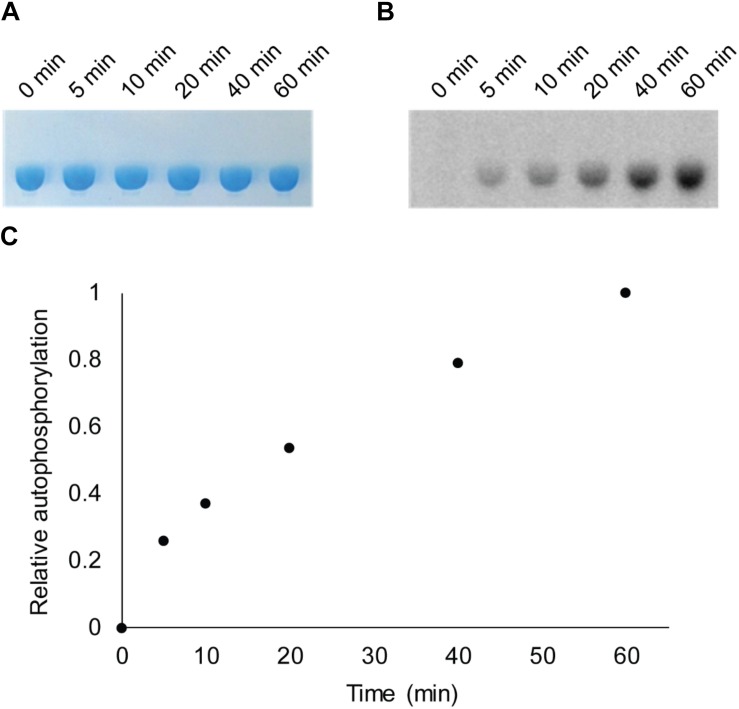
*In vitro* autophosphorylation activity assay of *Vp* HqsK (D499A). Autophosphorylation activity was detected by SDS-PAGE and autoradiography. **(A)** Stained polyacrylamide gel shows uniform protein loading. **(B)** Autoradiography shows increasing phosphate accumulation on *Vp* HqsK over time. **(C)** Quantification of autoradiography bands in **B**.

**FIGURE 5:**
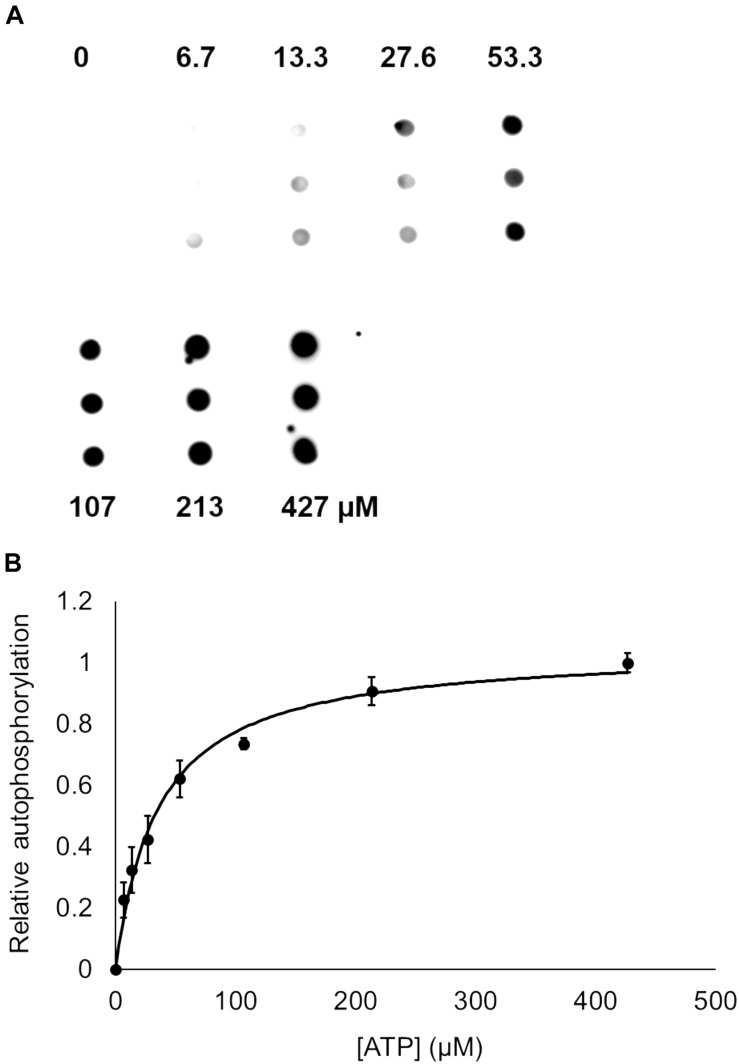
Dependence of *Vp* HqsK D499A autophosphorylation on ATP concentration. **(A)** Dot blot assay showing radiolabeled *Vp* HqsK D499A bound to a nitrocellulose membrane shows HqsK autophosphorylation follows Michaelis-Menten kinetics. Each concentration of ATP was assayed in triplicate using biological replicates. **(B)** Quantification of *Vp* HqsK D499A autophosphorylation as a function of ATP concentration. The apparent K_*m*_ is 35 μM. Error bars indicate the standard deviation of three biological replicates.

Bacterial *hnoX* genes often neighbor genes that code for signaling proteins, such as HKs, cyclic-di-GMP metabolizing proteins, or methyl accepting chemotaxis proteins (Iyer et al., 2003). In all H-NOX homologs characterized to date, H-NOX has been demonstrated to regulate the activity of its associated signaling protein as a function of binding to NO. In *V. parahaemolyticus*, *hnoX* is encoded in the same operon as a hybrid HK predicted to be involved in QS. We named this kinase HqsK (H-NOX-associated QS kinase). To identify whether HqsK (VP1876, gene ID: 1189383) is a functional kinase, we cloned, expressed, and purified the protein, and then conducted autophosphorylation activity assays. When HqsK is incubated with ATP containing trace [γ-^32^P]-ATP over time, the resulting autoradiography shows accumulating radiolabeled phosphate on the HK in a time-dependent manner, confirming kinase activity of *Vp* HqsK (Figure 4). We also tested the kinase activity as a function of ATP concentration (Figure 5). HqsK appears to follow Michaelis-Menten kinetics, with an apparent K_*m*_ of 35 μM.

**FIGURE 6:**
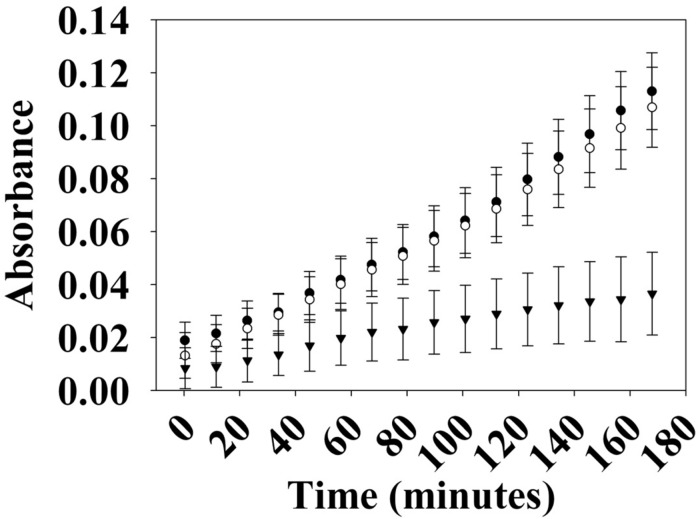
Phosphatase activity of *Vp* HK. Phosphatase activity over time was monitored using OMFP as a substrate (open circles, WT; closed circles, H214A; inverted triangles, D499A). *Vp* HK has phosphatase activity, which was diminished upon the mutation of conserved aspartic acid (D499A) in the kinase receiver domain. The plot shows the average of three independent assays ± SEM.

Many hybrid HKs are dual-functioning enzymes that have phosphatase activity in addition to kinase activity, in order to regulate the phosphorylation state of a partner response regulator (Parkinson and Kofoid, 1992). To test whether *Vp* HqsK is such an enzyme, phosphatase activity was monitored using a generic phosphatase substrate 3-O-methyl-fluorescein phosphate (OMFP) that yields O-methylfluorescein (OMF) upon dephosphorylation. Wild-type HqsK displays increasing OMF fluorescence over time, indicating *Vp* HqsK has phosphatase activity (Figure 6).

The kinase and phosphatase activities of hybrid HKs are dependent on conserved His and Asp residues, respectively (Kwon et al., 2000; Albanesi et al., 2009; Goodman et al., 2009). To demonstrate the importance of these residues for phosphatase activity, we generated H214A and D499A mutant HqsKs and repeated the phosphatase assay. Wild-type and H214A HKs have the same level of phosphatase activity as the wild-type enzyme, whereas D499A has significantly reduced phosphatase activity, indicating conserved D499 in the internal kinase receiver domain (IKR) is required for *Vp* HqsK phosphatase activity.

### *Vp* HqsK Transfers Phosphate to the Quorum Sensing Phosphotransfer Protein LuxU

**FIGURE 7:**
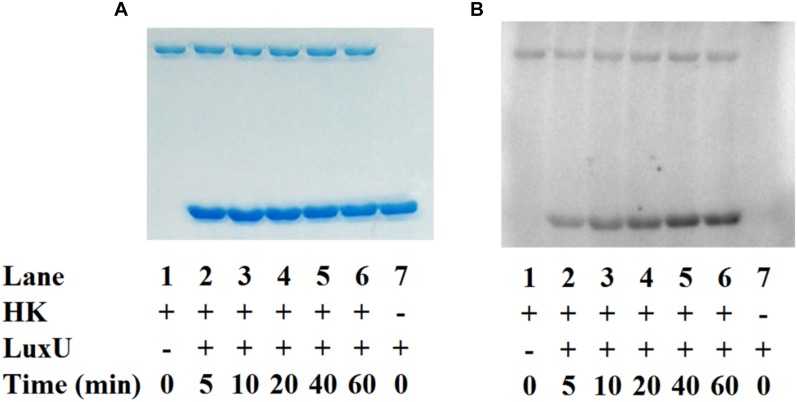
*In vitro* phosphotransfer assay of *Vp* HqsK to LuxU. Phosphotransfer activity was detected by SDS-PAGE and autoradiography. Stained polyacrylamide is shown for uniform protein loading **(A)**. *Vp* HqsK transfers phosphate to LuxU over time in the presence of ATP **(B)**. Time indicates the length of incubation after the addition of LuxU to HK.

**FIGURE 8:**
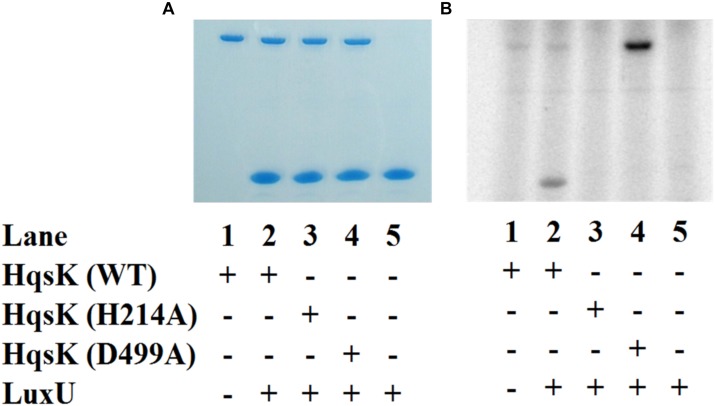
Phosphotransfer specificity test. *In vitro* phosphotransfer assay from HqsK to LuxU with WT, H214A, and D499A HqsK. Stained polyacrylamide gel **(A)** and autoradiography **(B)** are shown. H214A HqsK has no autophosphorylation activity due to the phosphorylation site mutation. D499A, kinase receiver domain mutant autophosphorylates, but does not phosphotransfer to receiver domain nor LuxU.

To establish the phosphotransfer circuit between *Vp* HqsK and the QS histidine-containing phosphotransfer protein LuxU, a phosphotransfer assay was conducted. Purified HqsK was incubated with ATP containing trace [γ-^32^P]-ATP, followed by the addition of purified LuxU. The resulting autoradiography shows an accumulation of radiolabeled phosphate on LuxU over time, indicating *Vp* HqsK transfers phosphate to LuxU (Figure 7). We also tested HqsK/LuxU phosphotransfer using the H214A and D499A HqsK mutants to confirm the transfer is occurring, as predicted, via phosphotransfer from H214 to D499 in HqsK followed by transfer to H56 in LuxU. As expected, H214A HqsK showed neither autophosphorylation nor phosphotransfer activities, whereas D499A HqsK exhibited only autophosphorylation activity (Figure 8).

### LuxU Is the Cognate Phosphotransfer Protein for *Vp* HqsK

*Vibrio parahaemolyticus* has three stand-alone Hpts. As described above, we have demonstrated that *Vp* HqsK transfers phosphate to LuxU, but since many HKs can transfer phosphate to multiple partners, there remained a possibility that this HqsK/LuxU phosphotransfer was not exclusive and/or that LuxU is not a cognate phosphotransfer partner for HqsK. It has been demonstrated that *in vitro*, an HK will have a kinetic preference for its *in vivo* cognate response regulator, exhibiting a much faster rate of phosphotransfer with its cognate partner (Laub et al., 2007). This preference is determined in an experiment called phosphotransfer profiling, in which either loss of phosphate from the HK or the appearance of a preferentially phosphorylated Hpt is detected using PAGE followed by autoradiography (Laub et al., 2007).

**FIGURE 9:**
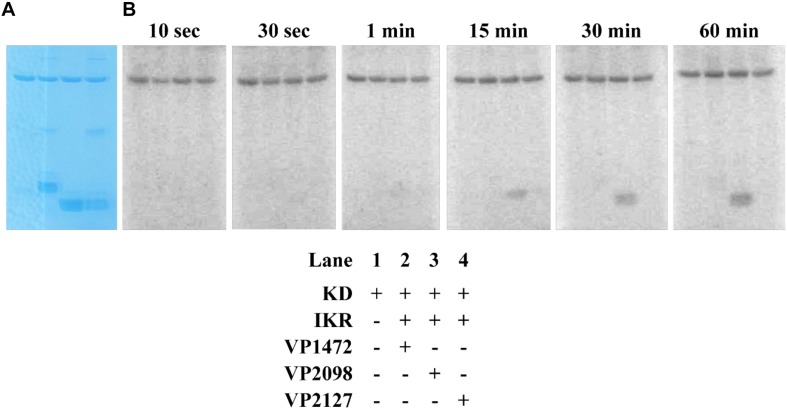
Phosphotransfer profiling of *Vp* HqsK and three stand-alone *Vp* Hpt proteins (VP1472, VP2098, and VP2127). Stained polyacrylamide gel **(A)** and autoradiography **(B)** are shown. 1 μM each of KD and IKR were incubated with 10 μM each of Hpts. KD/IKR were loaded with phosphoryl group by preincubating with ATP for 90 min. Hpt proteins were then added to initiate the phosphotransfer. Phoshoryl group accumulation was observed only for VP2098 (LuxU) among three Hpts.

**FIGURE 10:**
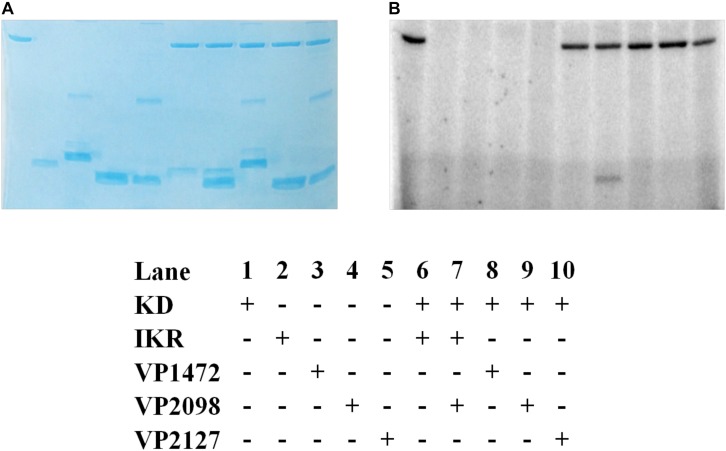
*Vp* HK, Hpt phosphotransfer specificity test. Various combinations of KD, IKR, and Hpt proteins were mixed to observe phosphotransfer. Stained polyacrylamide gel **(A)** and autoradiography **(B)** are shown.

The kinase domain and internal kinase receiver domain from *Vp* HqsK were separately cloned and expressed to isolate the kinase and receiver domains. We also purified all three stand-alone Hpt proteins from *V. parahaemolyticus*: LuxU (VP2098, gene ID: 1189609), VP1472 (gene ID: 1188978), and VP2127 (gene ID: 1189639). Then each Hpt was separately added to a mixture of *Vp* HqsK KD and IKR domains that had been preincubated with radiolabeled ATP. The resulting autoradiography showed no apparent band intensity loss from any of the kinase domains, nor band appearance for VP1472 or VP2098. However, a band corresponding to phosphorylated LuxU appears after 15 min and increases in intensity over time, indicating the accumulation of phosphate on LuxU (Figure 9). To verify this accumulation of phosphate on LuxU is not due to non-specific phosphorylation, but due to phosphotransfer through the HqsK KD/IKR domains, we conducted a phosphotransfer specificity test (Figure 10). The results indicate that LuxU does not phosphorylate itself, nor can it directly receive phosphate from the HqsK kinase domain, but it can only be phosphorylated via a His-Asp-His phosphotransfer through the HqsK kinase and IKR domains. Overall, these results show LuxU is the cognate phosphotransfer Hpt protein for HqsK.

### H-NOX Suppresses *Vp* HqsK Autophosphorylation Upon NO Binding

**FIGURE 11:**
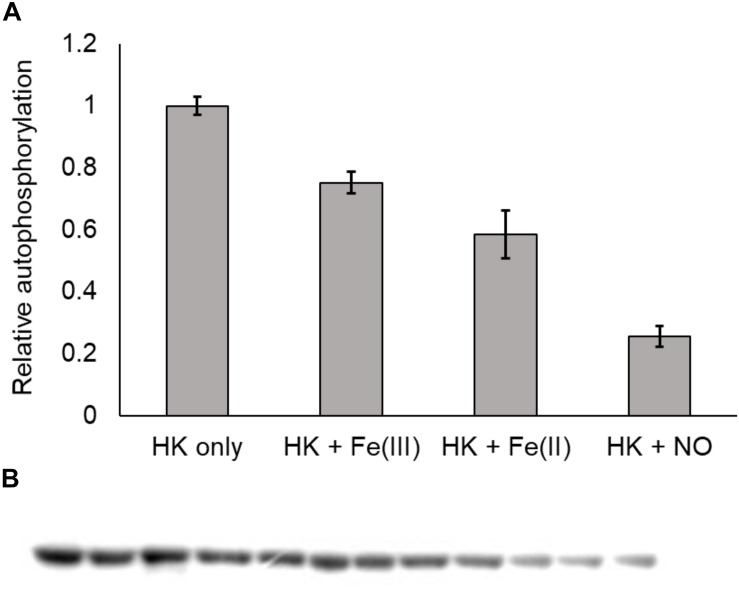
*Vp* H-NOX inhibits kinase activity of HqsK. **(A)** Quantification of HqsK autophosphorylation in the presence of various H-NOX complexes. Fe (II)-NO H-NOX displays the strongest inhibition of HqsK activity. Band intensity was quantified with ImageJ. **(B)** Autoradiography results showing relative levels of phosphorylated HqsK in the presence of each H-NOX complex. Each condition was assayed in triplicate. Lanes 1–3, HqsK only; lanes 4–6, HqsK + Fe (III) H-NOX; lanes 7–9, HqsK + Fe (II) H-NOX; lanes 10–12, HqsK + Fe (II)-NO H-NOX. Error bars indicate the standard deviation of three biological replicates.

**FIGURE 12:**
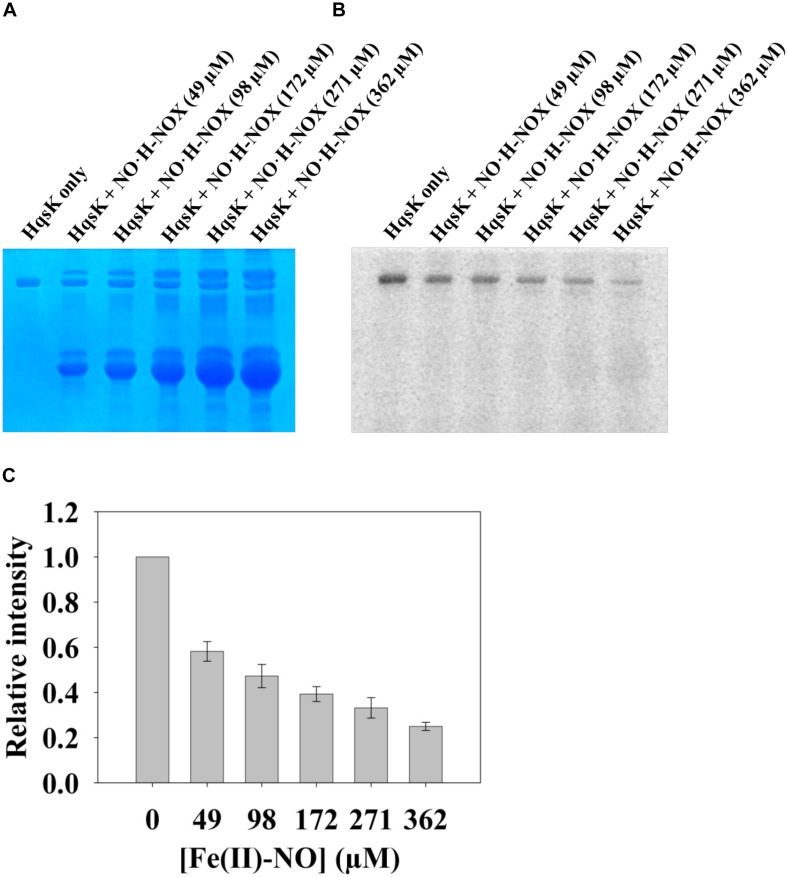
NO bound H-NOX inhibits HqsK kinase activity in a dose-dependent manner. *Vp* HqsK (D499A) was incubated with varying concentrations of NO⋅H-NOX for 30 min then another 30 min after the addition of ATP. Stained polyacrylamide gel **(A)** and autoradiography **(B)** are shown. **(C)** Quantified band intensities of HqsK titration with Fe^II^-NO H-NOX. Average of three independent assays ± SEM is plotted.

In the NO-responsive QS circuit in *V. harveyi*, the basal kinase activity of *Vh* HqsK was repressed by H-NOX upon NO binding (Henares et al., 2012). To test whether *Vp* HqsK is regulated in a similar manner, various ligation states of *Vp* H-NOX were incubated with HqsK and the effects on autophosphorylation were observed. *Vp* HqsK and Fe^II^-unligated, Fe^II^-CO or Fe^II^-NO *Vp* H-NOX were incubated in an anaerobic chamber, and then ATP with trace [γ-^32^P]-ATP was added to initiate HqsK autophosphorylation. The resulting autoradiography showed significant kinase activity inhibition with all *Vp* H-NOX complexes, with the most inhibition (75%) by Fe^II^-NO H-NOX (Figure 11). We also titrated HqsK with Fe^II^-NO H-NOX and observed decreasing *Vp* HqsK autophosphorylation in an Fe^II^-NO H-NOX concentration-dependent manner (Figure 12). Our data indicate that *Vp* H-NOX suppresses the kinase activity of the associated HqsK upon NO binding, similar to what has been observed in *V. harveyi*.

### The Effect of NO on the Master Quorum Regulatory Genes opaR and aphA

In the *V. parahaemolyticus* QS circuit, LuxU and LuxO ultimately regulate the translation of two master quorum regulatory proteins, OpaR, and AphA (Rutherford et al., 2011). However, these master quorum regulatory proteins have also been shown to reciprocally regulate each other’s gene transcription. AphA binds to the *luxR* promoter in *V. harveyi* and directly suppress transcription of *lux*R (Rutherford et al., 2011). In *V. parahaemolyticus*, OpaR binds to the *aphA* promoter and represses transcription of *aphA* (Zhang et al., 2012). These findings of transcriptional regulation of the master QS genes prompted us to investigate if NO has a role in regulating *aphA* and *opaR* transcription. Here, we analyze the effect of various concentrations of NO (delivered with the NO donor DETA NONOate) on the transcription of these master quorum regulatory proteins at low (A_600nm_ = 0.2) cell density using qRT-PCR.

**FIGURE 13:**
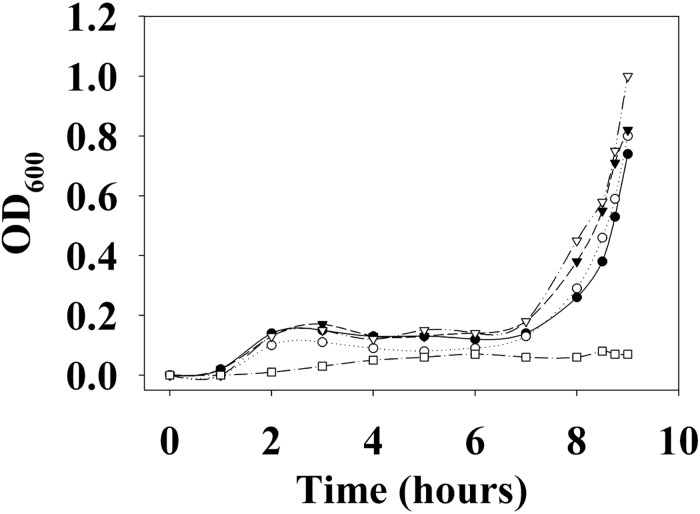
Growth curve of *V. parahaemolyticus*. *V. parahaemolyticus* was grown with 0 (closed circles), 50 (open circles), 100 (closed triangles), 200 (open triangles), and 500 μM (open squares) DETA NONOate (equivalent to 0, 190, 380, 760, and 1900 nM NO, respectively). Almost no growth was observed with 500 μM NONOate. For the detailed growth condition, see section “Materials and Methods.”

**FIGURE 14:**
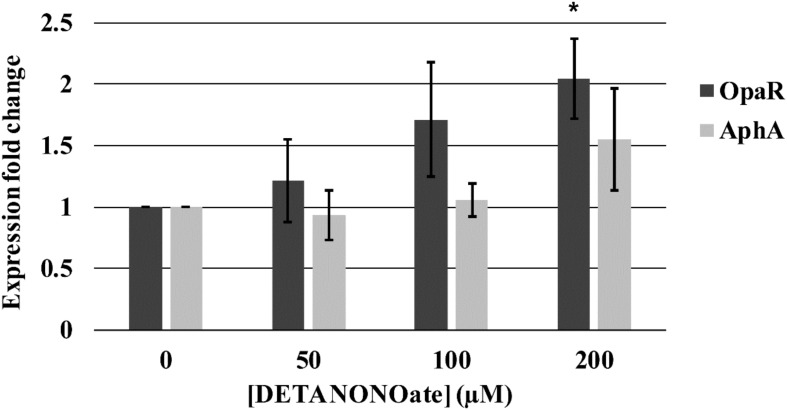
*Vp* master quorum regulatory protein transcription analysis by qPCR. Transcription of two *Vp* master quorum regulatory proteins, *aphA* and *opaR*, in the presence of various concentrations of DETA NONOate was analyzed. 0, 50, 100, and 200 μM NONOate are equivalent to 0, 190, 380, and 760 nM NO, respectively. ^∗^ denotes *p*-value < 0.05 compared to gene expression in 0 μM NONOate. Error bars represent SEM from four independent assays.

First, the growth of *V. parahaemolyticus* in the presence of various concentrations of DETA NONOate was monitored to determine the NO toxic threshold (Figure 13). The growth curve for cultures with 0, 50, 100, and 200 μM DETA NONOate [equivalent to 0, 190, 380, and 760 nM NO under these conditions (Bebok et al., 2002)] showed almost identical growth, but almost no cell growth was seen with 500 μM DETA NONOate (1900 nM NO). Accordingly, *V. parahaemolyticus* cultures grown with 0–200 μM DETA NONOate were used to monitor *aphA* and *opaR* transcription (Figure 14). The gene expression levels of *opaR* and *aphA* were quantified using *secY* (VP0277, gene ID: 1187744) as a reference gene.

We observed a trend of increasing transcription of *opaR* as a function of NO (1.22-, 1.71-, and 2.04-fold change with 50, 100, and 200 μM DETA NONOate, respectively). The fold-change of 2.04 with 200 μM NONOate was significantly different (*p* < 0.05) with respect to no NONOate, but the other two were not (*p* > 0.05). Transcript levels of *aphA* remained almost constant with increasing NO, although at 200 μM NONOate, there was an increase of 1.56 with respect to no NONOate.

An NO-dependent increase in both *aphA* and *opaR* transcription is unexpected since *opaR* suppresses *aphA* transcription. The mechanism of this increased *aphA* transcription at higher NO concentration is unknown. It is possible that although 200 μM NONOate did not hinder the bacterial growth, it is causing stress to bacteria, and increased *aphA* transcription is a part of the stress response. Also, it is possible that NO may be activating an unidentified signaling cascade that is turned on only at higher NO concentrations.

## Discussion

Quorum sensing is a key signaling system bacteria use to orchestrate population-wide gene expression patterns appropriate for different stages of growth or environmental conditions. Bacteria employ various types of QS circuits, AIs, and AI receptors that are most suitable for their particular lifestyle or the environment in which they live. Not only do bacteria monitor the AI concentrations of their own and other bacterial species, many actively interfere with QS circuits of competing species through a process called quorum quenching (Leadbetter and Greenberg, 2000; Dong et al., 2001). Some pathogenic or symbiotic bacteria also integrate host-organism-originated signals into QS to manage effective colonization and pathogenicity (Beck von Bodman et al., 1992; Fuqua and Winans, 1994; Hwang et al., 1994). Similarly, eukaryotes monitor bacterial AIs to detect their presence and activate an immune response (Givskov et al., 1996; Chun et al., 2004). Autoinducer-mediated QS is a dynamic communication system that spans across different bacterial species and even across kingdoms. Recent studies have revealed increasing complexity in QS signaling, indicating the growing importance of QS not only in the regulation of the bacterial life cycle, but also in our eukaryotic immune response.

Nitric oxide has been shown to promote bacterial biofilm dispersal through the regulation of the bacterial second messenger signaling molecule c-di-GMP in *Shewanella woodyi* (Liu et al., 2012) and *Shewanella oneidensis* (Plate and Marletta, 2012). Although their mechanisms have not been identified, NO has also been shown to modulate biofilm formation in *Pseudomonas aeruginosa* (Barraud et al., 2006) and *Nitrosomonas europaea* (Schmidt et al., 2004). In *V. harveyi* QS circuits, the NO signal is integrated into a QS signaling cascade through H-NOX/HqsK (Henares et al., 2012). H-NOX shifts HqsK’s activity from kinase to phosphatase upon NO binding. Together with other AI sensory HKs, it controls the phosphorylation state of the RR LuxO. LuxO ultimately regulates hundreds of gene expressions through two master quorum regulatory proteins LuxR (OpaR homolog) and AphA (Rutherford et al., 2011). Based on gene analysis, *V. harveyi* does not have an NO synthase. Thus, we hypothesize *V. harveyi* detects NO originated from its host organism or other NO-producing bacteria and integrates that information in its QS circuit. In this paper, we show that *V. parahaemolyticus* appears to also have an NO-responsive signaling pathway that feeds into the QS circuit.

The signaling cascade of *V. parahaemolyticus* NO-responsive QS circuit is similar to that of *V. harveyi*. NO is detected by a cytoplasmic H-NOX protein that switches the activity of HqsK between kinase and phosphatase. HqsK integrates the NO signal into a QS circuit to regulate the phosphorylation state of QS RR LuxO and ultimately control the transcription of two master quorum regulatory proteins OpaR and AphA. Studies on other *Vibrios* (*V. harveyi* and *V. cholerae*) show that low cell density promotes phosphorylation of LuxO and activation/suppression of *aphA*/*luxR* transcription. High cell density promotes dephosphorylation of LuxO and activation/suppression of *luxR*/*aphA* transcription. Our data suggest that NO functions as an AI and participates in the *V. parahaemolyticus* QS circuit. If OpaR and AphA’s reciprocal regulatory circuit is conserved in *V. parahaemolyticus*, we expect increasing NO concentration would promote *opaR* transcription and suppress *aphA* transcription. Our qPCR result supported the former part of the hypothesis. Increasing NO concentration promoted *opaR* transcription in a concentration-dependent manner, supporting a role for NO in the QS circuit as an AI. However, *aphA* transcription also increased at high NO concentration (200 μM). This discrepancy may be due to NO toxicity or may mean the presence of an unidentified NO signaling cascade in *V. parahaemolyticus*. We are currently investigating the mechanism behind the increased *aphA* transcription caused by NO and the alternative NO signaling cascade in *V. parahaemolyticus*.

## Data Availability

All datasets generated for this study are included in the manuscript and/or the supplementary files.

## Author Contributions

TU and JF performed the experiments. TU, JF, and EB wrote the manuscript.

## Conflict of Interest Statement

The authors declare that the research was conducted in the absence of any commercial or financial relationships that could be construed as a potential conflict of interest.
